# Selective Control Mechanisms, Quantitative Evaluation, and Sustainable Strategies for Cultural Heritage Surface Cleaning

**DOI:** 10.3390/polym18091116

**Published:** 2026-04-30

**Authors:** Jiaxin Zhang, Yutong Liu, Xiang Liu, Shanxiang Xu, Wenxuan Chen, Xinyou Liu

**Affiliations:** 1 College of Furnishing and Industrial Design, Nanjing Forestry University, Nanjing 210037, China; 3032751584@njfu.edu.cn (J.Z.); 2311404209@njfu.edu.cn (Y.L.); liuxiang@njfu.edu.cn (X.L.); xushanxiang@njfu.edu.cn (S.X.); wenxuanchen@njfu.edu.cn (W.C.); 2 Co-Innovation Center of Efficient Processing and Utilization of Forest Resources, Nanjing Forestry University, Nanjing 210037, China

**Keywords:** cultural heritage conservation, microemulsions, polymer gels, quantitative evaluation, selective cleaning, sustainable conservation

## Abstract

The conservation of cultural heritage artifacts requires precise and controlled cleaning strategies to remove surface contaminants while preserving the structural and aesthetic integrity of the original materials. Over time, artifacts made of stone, paper, textiles, and other materials are exposed to environmental pollution, chemical reactions, and microbial colonization, which lead to the accumulation of complex contaminant layers and progressive material degradation. In recent years, significant advances in materials science have introduced innovative cleaning approaches, including polymer gels, microemulsions, nanomaterials, and enzyme-assisted systems, which enable selective contaminant removal with reduced risk of substrate damage. These methods provide improved control over solvent release, contaminant dissolution, and interaction with sensitive surfaces compared to conventional mechanical and chemical cleaning techniques. In addition, advanced analytical tools such as Raman spectroscopy, surface-enhanced Raman spectroscopy (SERS), and X-ray fluorescence (XRF) have enabled quantitative evaluation of cleaning efficiency and more accurate monitoring of conservation processes. This review summarizes the major contamination mechanisms affecting cultural heritage materials and discusses recent developments in cleaning technologies, functional materials, and evaluation methods. The analysis shows that selective cleaning methods can significantly minimize damage to the underlying substrate, while environmentally friendly functional materials combined with multi-dimensional quantitative evaluation provide an effective and sustainable framework for cultural heritage conservation.

## 1. Introduction

Cultural heritage represents a vital repository of human history, art, and science, and its integrity and aesthetic value are indispensable for societal development and cultural transmission. However, over time, environmental factors and human activities increasingly impact heritage objects. Stone artifacts, due to prolonged exposure to outdoor conditions, are particularly vulnerable to natural weathering, air pollution, and physical contact, which can result in surface accumulation of dust, oils, salts, and microbial colonies, ultimately causing discoloration, corrosion, and crack propagation [1]. Similarly, paper-based artifacts, calligraphy, and murals are susceptible to damage from light, humidity, microbial activity, and acidic compounds during preservation, leading to fiber degradation, pigment fading, and loss of mechanical strength [2,3]. Metal artifacts are also susceptible to corrosion processes driven by environmental moisture, pollutants, and surface contaminants, which can accelerate the formation of corrosion layers and compromise the stability of historical materials [4].

The cleaning of surface contaminants is a critical and irreversible step in the conservation and restoration of cultural heritage. Because cleaning directly interacts with the original surface of artifacts, inappropriate treatments may permanently remove historical information or alter the physicochemical properties of the substrate. Effective cleaning not only involves the removal of dust, coatings, old restoration materials, oils, microbes, and other pollutants but also requires the preservation of the physical and chemical stability of the underlying substrate [5]. Different materials exhibit varying sensitivities to contaminants and cleaning treatments due to their distinct physical, chemical, and biological properties [1,2,3,4,5]. For instance, porous stone materials may retain soluble salts and microbial biofilms within their microstructures; metals are susceptible to corrosion reactions during aqueous cleaning processes; while paper and textile artifacts possess fragile fibrous networks that can be easily damaged by aggressive solvents or mechanical stress [4,6,7,8,9,10]. Therefore, cleaning methods must be precisely selected—considering not only the chemical composition of the contaminant but also the fragility and degradation state of the substrate—to achieve an optimal balance between cleaning efficacy and material preservation. Traditional cleaning approaches, including mechanical, chemical, and aqueous methods, often risk secondary contamination or damage to the artifact. In recent years, sustainable and environmentally friendly approaches have gained attention, such as enzymatic cleaning, microemulsion-loaded gels, hydrogels, and nanocomposite systems. These methods maximize the removal of contaminants while preserving the original structure and aesthetic of the artifact [11,12,13]. Recent advances in nanostructured fluids, polymeric gels, and bio-based solvents have further improved the selectivity and safety of cleaning treatments, particularly for fragile substrates [9,10].

This review focuses on stone, metal, paper, and textile artifacts, which represent four major categories of cultural heritage materials most frequently subjected to conservation cleaning. These materials represent a major portion of museum and archaeological collections. They exhibit diverse contamination and degradation mechanisms that require tailored cleaning strategies. Wall paintings and ceramics are excluded from this review due to their distinct conservation methodologies and the availability of specialized literature [12].

Microbial biodeterioration poses an additional threat to cultural heritage materials, as microorganisms can induce surface deterioration through acidic metabolic byproducts, mineral dissolution, and biofilm formation, collectively accelerating discoloration and structural degradation [1,3,6,7,8,14]. In recent years, sustainable cleaning strategies such as enzyme-based systems, microemulsion-loaded gels, hydrogels, and nanostructured fluids have gained increasing attention in cultural heritage conservation [9,10,11,12,13]. These systems enable selective and controllable removal of contaminants, including oils, adhesives, microbial growth, and aged surface deposits, while minimizing penetration into fragile substrates and preserving original material integrity. Combined with advanced analytical techniques such as Raman spectroscopy, SERS, and XRF, cleaning performance can be quantitatively evaluated at micro- and molecular scales, enabling more precise and scientifically guided conservation decisions [11,15].

**Figure 1:**
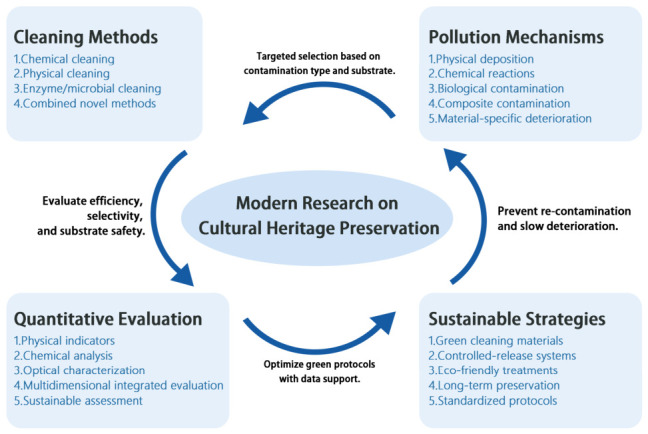
Research framework of this review.

This review systematically addresses cleaning mechanisms, material applications, contamination control, quantitative evaluation methods, and sustainable practices, providing a scientific basis and methodological reference for future heritage conservation research. To provide a clear overview of the scope and structure of this review, the overall research framework is illustrated in Figure 1.

## 2. Surface Materials of Cultural Heritage and Contamination Mechanisms

**Figure 2:**
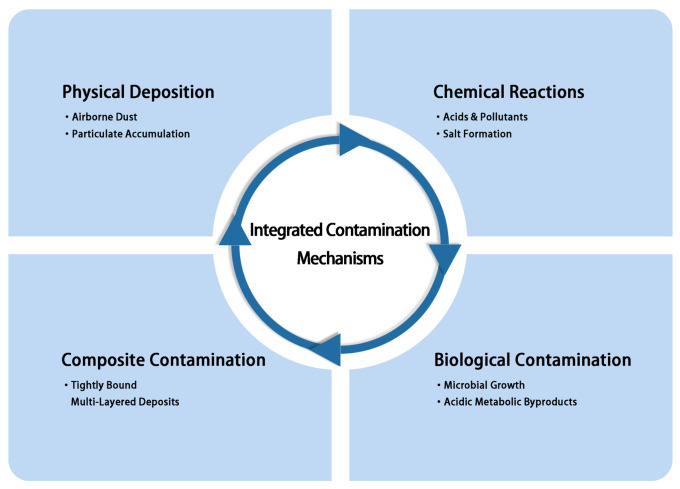
Classification of sources of contaminants on cultural relics.

Surface contamination is a central concern in the preservation of cultural heritage. The contamination mechanisms are multifaceted, reflecting the interplay of physical, chemical, and biological factors. These mechanisms often coexist and may reinforce one another—for example, dust deposition facilitates microbial colonization, while salt formation accelerates microbial degradation—resulting in multilayered contaminant structures that increase cleaning complexity and challenge substrate integrity [16]. Understanding these integrated mechanisms is essential for selecting appropriate cleaning methods and developing scientific conservation strategies [1,13]. Based on these mechanisms, contamination sources can be categorized into four interrelated types according to their origin and action, as illustrated in Figure 2: physical deposition, chemical reactions, biological contamination, and composite contamination.

The type and extent of contamination on artifacts vary significantly depending on material composition and environmental exposure, reflecting the interplay of physical, chemical, and biological factors. Prolonged exposure to natural or anthropogenic environments leads to the accumulation of airborne particles, dust, oils, salts, and microbial colonies, forming multilayered contamination structures that contribute to progressive material degradation. These processes are governed not only by environmental parameters such as humidity, temperature, light exposure, and atmospheric pollutants, but also by the intrinsic physical structure, chemical composition, and biological activity of the artifacts. Understanding these mechanisms is essential for developing targeted cleaning strategies and ensuring the long-term stability of cultural heritage materials. Although their manifestations vary across different heritage materials, these contamination processes are universally governed by coupled physical-chemical-biological interactions.

### 2.1. Stone Artifacts

Stone artifacts encompass historical steles, sculptures, walls, and various stone-built structures, whose composition and microstructure depend on geological origin and fabrication techniques. Stone typically exhibits high porosity and capillary networks, making it highly susceptible to the adsorption of airborne particulate matter, dust, microbial spores, and acidic gases [1]. The extent and nature of contamination vary significantly depending on the stone type: porous stones such as limestone and marble are more susceptible to salt crystallization and acid attack, while dense stones such as granite are more resistant but may still accumulate surface deposits.

**Figure 3:**
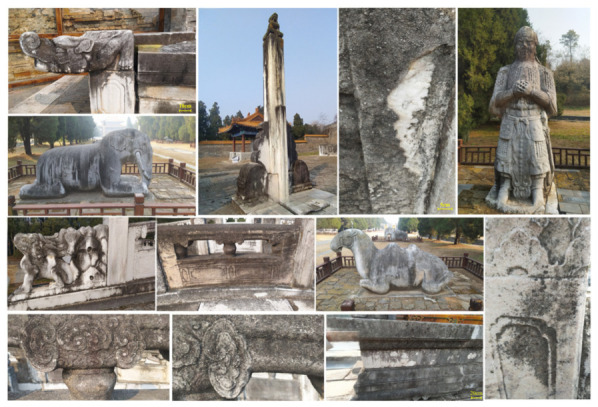
Numerous stone artifacts from the Xianling Tomb, featuring black stains on their surfaces [15].

A particularly common and complex type of soiling on stone artifacts is black crust formation. Black crusts typically consist of gypsum (calcium sulfate dihydrate) formed by the reaction of sulfur dioxide (SO_2_) from air pollution with calcium carbonate (CaCO_3_) in the stone. The formation mechanism involves three interacting processes: (i) dry and wet deposition of atmospheric pollutants (SO_2_, NO_x_, and particulate matter) onto the stone surface; (ii) microbial metabolic activity that produces organic acids, which may enhance mineral dissolution and accelerate crust formation; and (iii) environmental factors such as humidity, temperature, and rain patterns that influence the rate and distribution of crust development [1,13,15]. Black crusts typically consist of particulate matter, metal oxides, and microbial metabolic by-products, forming tightly adhered layers that are difficult to remove, as shown in Figure 3.

The black stains visible in Figure 3 correspond to black crust formation on stone surfaces, a common deterioration phenomenon driven by atmospheric pollution and surface chemical reactions. These crusts are primarily composed of gypsum, particulate matter, metal oxides, and microbial residues, forming compact and strongly adhered layers on the stone surface. Due to their adhesion and partial penetration into the pore network, such crusts are difficult to remove without affecting the underlying substrate, making selective cleaning a major conservation challenge [1,13].

For stone artifacts, typical contamination types include salt crystallization (from weathered mineral dissolution and atmospheric deposition), black crust formation (from chemical reactions with atmospheric pollutants), and microbial biofilm deposition [1,13]. The cleaning challenges associated with stone artifacts are primarily related to their porous structure, which allows contaminants to penetrate deeply, and the strong adhesion of crusts to the underlying substrate.

Microorganisms represent a critical factor in the biodeterioration of stone artifacts. Bacteria, fungi, and algae colonize pores, secreting organic acids, enzymes, and other metabolites that dissolve minerals and disrupt microstructures [14]. Residual microorganisms or their metabolites may reactivate under favorable environmental conditions (e.g., increased humidity or temperature), leading to biofilm regrowth and renewed degradation. Porous stone structures may retain spores deep within their microstructure, making complete eradication difficult and necessitating ongoing monitoring and preventive conservation strategies [4,6,7,8].

The cleaning of stone artifacts will be discussed in detail in Section 3 of this review.

### 2.2. Metal and Alloy Artifacts (Inorganic Material)

Metal artifacts develop oxide layers, carbonates, and other surface deposits through long-term exposure to environmental factors such as humidity, oxygen, and atmospheric pollutants [17]. Airborne dust and pollutants readily adhere to these surfaces, forming complex contamination layers that accelerate corrosion and compromise aesthetic and structural integrity [13].

Typical contamination types on metal artifacts include oxide layers (e.g., rust on iron), carbonates and chlorides (e.g., malachite and atacamite on bronze), and sulfides (e.g., silver tarnish). The cleaning of metal artifacts presents specific challenges: corrosion products may be either protective or destructive depending on their composition and stability; aggressive cleaning can remove desirable patina layers; and incomplete removal of chlorides can lead to post-cleaning corrosion recurrence [18].

Biofilm formation and microbial colonization also contribute to the deterioration of metal artifacts. Fungi and sulfur-oxidizing or nitrate-reducing microorganisms can secrete organic acids, create corrosive microenvironments, and promote electrochemical corrosion processes. Long-term microbial colonization may lead to recurrent corrosion even after initial cleaning, as microbial residues or biofilms can reactivate under suitable environmental conditions [7].

The cleaning methods for metal artifacts, including chelator-loaded hydrogels, laser cleaning, and pH-responsive gels, will be discussed in detail in Section 3 of this review [18,19,20].

### 2.3. Paper-Based Artifacts (Organic Material)

Paper-based artifacts include historical books, rice-paper paintings, archival documents, and manuscripts. Their primary constituents are cellulose, hemicellulose, and adhesives, with some containing mineral fillers or sizing layers to enhance strength and visual properties. These artifacts are highly sensitive to environmental factors such as light, humidity, temperature fluctuations, and airborne pollutants, which accelerate fiber degradation and reduce material performance.

For paper artifacts, typical contamination types include dust deposition, fungal growth, metal ion accumulation, and organic residue adherence [21].

A critical chemical contamination mechanism in paper artifacts is acid-induced degradation. The pH of paper directly influences its stability: cellulose is susceptible to acid-catalyzed hydrolysis, which breaks the glycosidic bonds between glucose units, leading to depolymerization and loss of mechanical strength. Acidic compounds can originate from multiple sources: (i) atmospheric pollutants such as sulfur dioxide and nitrogen oxides that react with moisture to form acids; (ii) microbial metabolic by-products, including organic acids produced by fungi and bacteria; (iii) degradation products of cellulose and hemicellulose themselves, such as formic and acetic acids; and (iv) historical manufacturing processes that may have left residual acids in the paper [22,23,24,25]. The resulting decrease in pH creates an autocatalytic degradation cycle, where acid hydrolysis produces more acidic compounds, further accelerating fiber breakdown. This process leads to embrittlement, yellowing, and eventual loss of structural integrity [6,8].

Fungi and bacteria are major biological contaminants on paper. In high-humidity environments, fungi can rapidly colonize and secrete extracellular enzymes that partially degrade cellulose and adhesives, leading to embrittlement, fiber breakage, localized discoloration, and reduced tensile strength [22,23]. Fungal metabolic by-products, including organic acids, may further alter surface pH, accelerating degradation [24,26].

Residual microbial spores or enzymes may remain viable after cleaning and can reactivate under favorable humidity and temperature conditions, leading to recurrent biodeterioration. Additionally, cleaning treatments that alter the pH of paper (e.g., aqueous washing) may affect its long-term chemical stability, requiring careful consideration of treatment parameters and post-treatment storage conditions [6,8].

**Figure 4:**
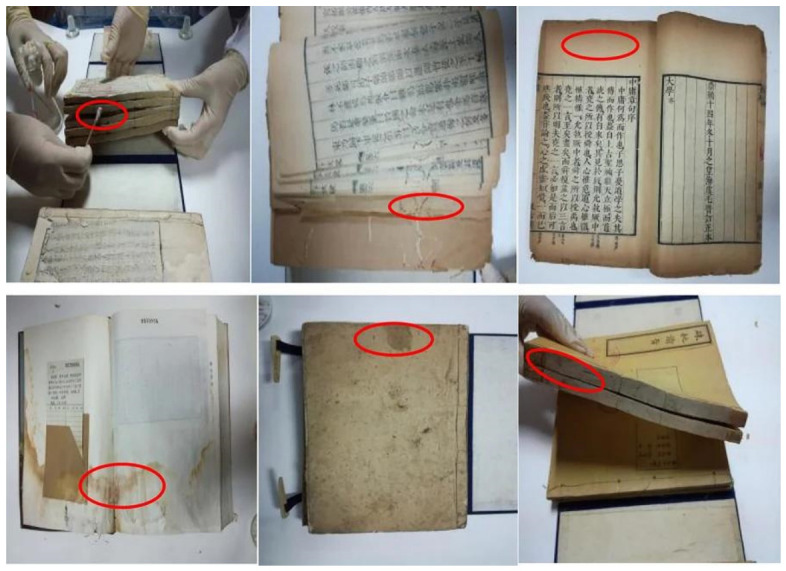
Ancient books with contaminants in the Special Collection Room of Liaoning University (red circles indicate the locations of the contaminants) [21].

Surface coatings or sizing layers often create microporous gradients that allow contaminants to penetrate the fiber matrix, increasing cleaning difficulty and raising the risk of secondary damage during treatment [27]. Additionally, poor ventilation, high moisture, or fluctuating temperatures exacerbate biological contamination, while interactions with airborne metal ions and organic compounds can produce persistent, hard-to-remove deposits. The microstructural features of paper, including fiber alignment, porosity, and coating thickness, further determine contaminant distribution patterns and complexity, highlighting the need for carefully tailored cleaning strategies. Figure 4 illustrates fungal growth and contamination distribution on a paper artifact surface, visually demonstrating the relationship between biological pollution and fiber structure [21].

The cleaning methods for paper artifacts, including enzymatic cleaning, microemulsion-loaded hydrogels, and aqueous gel systems, will be discussed in detail in Section 3 of this review [10,28,29].

### 2.4. Textile Artifacts (Organic Material)

Textile artifacts, including silk, cotton, wool, and blended fabrics, represent essential elements of traditional garments, decorations, and ritual objects. Their loose fiber networks, high surface area, and hygroscopic nature make them highly prone to adsorption of oils, dust, and organic stains and microbial colonization [30,31].

The main contamination types on textiles include particulate deposition (dust and soils), microbial colonization (fungi and bacteria), organic residues (oils, fats, and adhesives), and stains from dyes and coloring agents [22,30].

Fiber type, weave density, and dyeing processes influence both contaminant adhesion and penetration: smooth silk surfaces accumulate oils superficially, whereas porous cotton and linen allow water-soluble contaminants to infiltrate fibers, and the scaly surface of wool captures small particulates and spores. The hygroscopic nature of textiles also facilitates the absorption of moisture, creating a favorable environment for microbial growth and enzymatic degradation of fibers [22].

Microbial colonization under humid or warm conditions leads to enzyme-mediated degradation of cellulose or proteins, resulting in fiber embrittlement, strength loss, localized discoloration, and odors [22]. Fungi such as Aspergillus and Penicillium secrete cellulases and proteases that break down cellulose and protein fibers, while bacteria like Bacillus and Streptomyces produce a range of hydrolytic enzymes capable of degrading textile components [22,23]. The metabolic by-products of these microorganisms, including organic acids, can further alter the pH of the fiber environment, accelerating chemical degradation and weakening the textile structure.

Long-term exposure after initial soiling can lead to recurrent contamination. Residual oils or microbial metabolites may continue to interact with fibers, causing progressive discoloration or weakening even after cleaning. Furthermore, incomplete removal of microbial spores can result in regrowth under suitable environmental conditions, leading to secondary biodeterioration [6,8].

Prolonged deposition of oils, dust, and dyes forms complex contaminant layers that chemically interact with fibers, leading to insoluble residues and mechanical weakening. Oily contaminants penetrate amorphous fiber regions, reducing strength, while particulate matter becomes embedded within fiber networks, complicating removal [30]. Additionally, dye molecules may undergo chemical reactions with contaminants, leading to color changes and further complicating conservation efforts.

The cleaning methods for textile artifacts will be discussed in detail in Section 3 of this review [31,32,33].

## 3. Methods for Surface Cleaning of Cultural Heritage Artifacts

Surface cleaning of cultural heritage artifacts is a critical component of conservation and restoration, with the primary goal of removing surface contaminants, aged coatings, and residues from previous interventions while preserving the integrity and structural stability of the underlying materials [13,24]. Based on the underlying cleaning mechanisms and the types of artifacts, existing approaches can be broadly categorized into chemical cleaning, physical cleaning, and enzyme/microbial-based cleaning. Table 1 summarizes common surface cleaning methods, their typical applications, mechanisms of action, advantages, and limitations.

### 3.1. Chemical Cleaning Methods

Chemical cleaning primarily employs solvents or active surfactants to dissolve, emulsify, or chemically react with contaminants, making it suitable for oil-based, resinous, or polymeric coatings [13,34]. In addition to traditional solvent systems, recent developments have significantly expanded the range of chemical cleaning media.

Traditional organic solvents include ethanol, acetone, toluene, and petroleum ether, which are effective for removing waxes, oils, and aged varnishes. However, their use is associated with risks such as substrate swelling, pigment leaching, and exposure to volatile organic compounds (VOCs). Therefore, careful optimization of concentration, contact time, and application mode is essential for safe application.

Green solvents have emerged as sustainable alternatives, including bio-based solvents (e.g., ethyl lactate, linalool, and limonene), supercritical CO_2_, and deep eutectic solvents (DESs). These systems offer reduced toxicity and improved environmental compatibility. For example, linalool-based nanostructured fluids have demonstrated high efficiency in removing alkyd paints with minimal residue formation [10,13,45,46,47], while ethyl lactate provides biodegradable and low-toxicity cleaning performance suitable for paper and textile artifacts.

Supercritical CO_2_ cleaning enables the dissolution of organic contaminants without leaving chemical residues, making it particularly suitable for complex geometries such as metal ornaments and carved wooden objects. Solvent vapor cleaning provides a controlled alternative in which solvent vapors condense on the artifact surface, enabling gradual swelling and detachment of contaminant layers while minimizing liquid penetration into porous substrates.

Importantly, although vapor-phase and supercritical systems are discussed here due to their solvent-based mechanisms, they are also conceptually linked to controlled delivery systems and may be considered as transitional approaches between conventional chemical cleaning and advanced hybrid systems.

Microemulsions and nanostructured fluids (NFs) are widely applied for the removal of both hydrophilic and hydrophobic contaminants. Their mechanism involves solvent penetration into the contaminant layer followed by swelling and interfacial tension reduction, promoting detachment [35]. Although different formulations may vary in composition, the underlying mechanism consistently relies on interfacial energy modulation and controlled swelling of the contaminant layer.

Microemulsions are thermodynamically stable systems composed of water, oil, surfactant, and cosurfactant, forming nanoscale droplets. When incorporated into hydrogels, they allow controlled delivery of cleaning agents, reducing evaporation and lateral spreading [5]. Switchable surfactants have further enhanced these systems by enabling pH- or temperature-responsive emulsification and demulsification, facilitating residue-free removal [36].

Residual solvents or surfactants may remain within porous substrates such as stone, paper, and textiles, potentially causing delayed effects. For instance, surfactant residues may increase hygroscopicity and promote microbial growth on stone surfaces, while solvent retention in paper fibers may accelerate acid-catalyzed hydrolysis. Therefore, post-treatment removal and formulation optimization are essential.

In situ monitoring techniques, such as quartz crystal microbalance with dissipation (QCM-D), enable real-time tracking of polymer removal processes, supporting the optimization of cleaning parameters during treatment [35].

### 3.2. Physical Cleaning Methods

Physical cleaning relies on mechanical, optical, or acoustic means to detach contaminants and represents an important non-chemical approach. Common techniques include laser cleaning, ultrasonic cleaning, and micro–nano-bubble (MNB) cleaning.

**Figure 5:**
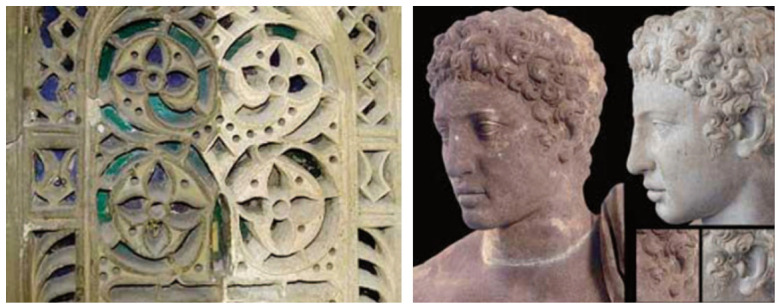
Comparison of stone cultural relics before and after laser cleaning. The left image shows the marble surface with black crusts and pollutants; the right image shows the cleaned surface.

Laser cleaning interacts with both the contaminant layer and the substrate to vaporize or lift the pollutant while preserving the underlying material [37,38], as shown in Figure 5.

The efficiency of laser cleaning depends on wavelength, pulse duration, and fluence. Nanosecond and femtosecond laser systems allow highly controlled ablation with minimized thermal damage. For sensitive artifacts such as painted surfaces or metallic artifacts, Nd:YAG or Er:YAG lasers enable selective removal of aged coatings [18].

Although laser cleaning is a non-contact and residue-free method, its long-term effects on substrate stability must still be considered. In some cases, localized thermal accumulation may induce microstructural changes such as micro-cracking or surface roughness modification, which may influence subsequent environmental interactions. These effects are generally dependent on laser parameters and material properties and are not universally observed.

Ultrasonic cleaning utilizes cavitation to detach contaminants [39]. The implosion of cavitation bubbles generates localized shock waves and micro-jets that dislodge particles from surfaces. Ultrasonic cleaning can be combined with microbubbles or gels to enhance cleaning efficiency while minimizing mechanical stress on fibers or fragile substrates [31,33]. The combination of low-frequency ultrasound (20–40 kHz) with stabilized microbubbles has been shown to improve cleaning efficacy on cellulose-based materials by increasing cavitation activity near the substrate surface [33].

Although both ultrasonic cleaning and MNB systems involve bubble-related phenomena, their generation mechanisms are fundamentally different: ultrasonic cavitation is induced by acoustic pressure fluctuations, whereas MNBs are pre-formed stable bubbles that collapse under physical or chemical stimuli.

This distinction leads to different operational behaviors: ultrasonic cleaning primarily provides mechanical removal, while MNB systems combine mechanical and chemical oxidative effects.

Micro–nano-bubble (MNB) cleaning relies on the generation and collapse of stable bubbles ranging from nanometers to micrometers, producing reactive oxygen species (ROS) such as hydroxyl radicals that chemically oxidize organic contaminants [40].

The impact of both methods depends strongly on the substrate type. On hard materials such as metals and ceramics, cleaning efficiency is high with minimal damage. However, on fragile materials such as paper and textiles, repeated cavitation or bubble collapse may gradually weaken fiber structures. Therefore, parameter optimization and post-treatment stabilization are necessary.

### 3.3. Enzyme and Microbial Cleaning

Enzyme-based cleaning exploits the catalytic hydrolysis of specific chemical bonds to remove protein, lipid, and resin contaminants. The high substrate specificity of enzymes enables selective removal of targeted contaminants without damaging the underlying artifact materials [30,41].

Enzyme immobilization has emerged as a key strategy for improving the practical applicability of enzyme cleaning. By confining enzymes within gel matrices, it is possible to achieve localized enzyme delivery, prevent enzyme leaching, and facilitate easy removal after treatment. Enzyme agarose gels incorporating pancreatic protease and yeast lipase have been developed for the selective hydrolysis of proteinaceous and lipid contaminants under mild conditions (pH 7–8, temperature 25–40 °C), effectively removing overpaint layers from historical paintings without damaging the original pigments or varnishes [41].

Proteases (e.g., trypsin, chymotrypsin, and papain) are effective for removing protein-based stains such as blood, egg, milk, and collagen-based adhesives. Lipases target lipid and oil residues, including drying oils and waxes commonly found in coatings and restoration materials [41]. Cellulases can selectively degrade cellulose-based stains or facilitate the removal of paper residues from textiles and other substrates [22,23]. Amylases are employed to remove starch-based adhesives and sizing agents [42].

Microbial cleaning utilizes whole microorganisms to degrade complex contaminant mixtures. Bacteria such as Pseudomonas, Bacillus, and Streptomyces species have been successfully employed to remove graffiti, overpaint, and biological stains from stone, wall paintings, and textiles [14,43,44]. The advantages of microbial cleaning include the ability to degrade multiple contaminant types simultaneously and the potential for self-sustaining treatment through biofilm formation. However, careful control of microbial growth conditions is required to prevent unintended biodeterioration of the substrate.

Residual enzyme activity or viable microorganisms may persist after treatment and may reactivate under favorable humidity and temperature, potentially leading to secondary biodeterioration. For example, residual proteases may continue degrading gelatin sizing in paper, while surviving microbial spores may germinate and form resistant biofilms. Similarly, bacterial spores that survive cleaning can germinate under humid conditions, forming new biofilms that are even more resistant to subsequent treatments [6,7,8].

To mitigate these risks, controlled-release enzyme gels have been developed, where enzymes are encapsulated within polymer matrices (e.g., agarose, alginate, and polyacrylamide) that gradually release active enzymes during cleaning and can be completely removed afterward. These systems minimize enzyme residues and allow precise control over reaction time and intensity. For microbial cleaning, post-treatment deactivation protocols (e.g., ethanol rinses, heat treatment, or UV irradiation) should be implemented to ensure that no viable microorganisms remain. Long-term monitoring studies have demonstrated that properly deactivated microbial treatments pose no greater risk than conventional cleaning methods, but incomplete deactivation can lead to recurrent biodeterioration within 6–12 months [14].

Enzyme and microbial methods are environmentally friendly and gentle but generally act more slowly than chemical or physical methods, making them suitable for localized cleaning or in combination with other techniques. Recent advances have focused on developing smart enzyme-responsive systems that release active enzymes only upon contact with specific contaminants, further improving selectivity and safety.

### 3.4. Combined and Novel Methods

Hybrid approaches are increasingly employed, such as integrating gels with nanofluids for localized fixation and controlled release, minimizing substrate infiltration [46,47]. The combination of microgels with nanostructured fluids allows the formation of stable emulsions that can be applied as a paste or spread, providing intimate contact with the contaminant layer while preventing solvent spreading into unprocessed areas [46].

Combined systems may reduce re-treatment frequency by maintaining controlled release and minimizing contaminant redeposition. This is particularly beneficial for removing thick or aged contaminant layers that require prolonged solvent contact. Controlled-release systems also reduce the risk of contaminant redeposition by maintaining stable solvent concentrations at the cleaning interface, preventing the reattachment of dissolved pollutants. Consequently, combined methods often result in more durable cleaning outcomes with lower recurrence rates compared to single-technique approaches [5,46].

Wei et al. proposed combining gels with ultrasonic emulsification for targeted textile cleaning, achieving precise, controllable stain removal [32]. This synergistic approach leverages the mechanical action of ultrasound to enhance gel penetration into fibrous substrates while maintaining the controlled release properties of the gel matrix.

For outdoor coatings or oily deposits, natural green solvents (e.g., linalool, limonene, and terpenes) combined with microemulsions or microcapsules can effectively remove stubborn contaminants while preserving substrate integrity. Linalool-based nanostructured fluids have been specifically formulated for the removal of alkyd paints, demonstrating high cleaning efficiency with minimal residue [47].

Emerging trends include the development of photoresponsive gels that undergo sol–gel transitions upon light irradiation, enabling remote-controlled cleaning with spatiotemporal precision. Electrochemically switchable surfactants and magnetic nanoparticle-assisted cleaning are also being explored for targeted contaminant removal in complex geometries.

Modern artifact cleaning methods are evolving toward highly selective, low-impact, and material-friendly strategies. By precisely controlling chemical, physical, and biological mechanisms, customized cleaning protocols can be developed for different contaminant types, providing sustainable conservation solutions for cultural heritage.

## 4. Quantitative Evaluation Methods for Surface Cleaning of Cultural Heritage Artifacts

In the conservation of cultural heritage, surface cleaning of artifacts not only aims to remove contaminants but also to preserve the integrity and structural stability of the underlying materials. Establishing a scientific and quantitative evaluation framework is therefore crucial for optimizing cleaning protocols and ensuring their sustainable application [48,49]. A key distinction must be made between qualitative and quantitative evaluation methods. Qualitative methods (e.g., visual inspection, FTIR peak identification, optical microscopy) provide descriptive information about cleaning outcomes, while quantitative methods generate numerical data that can be statistically analyzed and compared across treatments. This section focuses primarily on quantifiable indicators, including physical measurements (e.g., surface roughness, microcrack width, fiber tensile strength), chemical kinetics (e.g., QCM-D mass removal rates), and colorimetric data (ΔE values). Optical observations are retained where they provide semi-quantitative or visual context, but they are clearly distinguished from strictly quantitative indicators.

Modern evaluation approaches are typically classified into three main categories: physical measurements, chemical analyses, and optical characterization. By combining traditional techniques with high-precision instrumentation, a multidimensional and systematic assessment of cleaning performance can be achieved.

### 4.1. Physical Indicators (Quantifiable)

Physical evaluation focuses on assessing the structural integrity and mechanical performance of artifact surfaces. These measurements produce numerical values that enable objective comparison of cleaning protocols.

For stone artifacts, measurements such as surface roughness (Ra, Rq, Rz in micrometers), microcrack width (measured via SEM or optical profilometry), and porosity percentage provide insights into potential mechanical damage during cleaning. Non-destructive techniques such as 3D optical profilometry and X-ray microtomography (micro-CT) enable high-resolution characterization of surface topography and internal pore networks before and after cleaning, allowing quantitative assessment of cleaning-induced alterations [50].

For paper-based and fibrous artifacts, tensile strength (N/mm^2^), fiber durability (cycles to failure), and elongation at break (%) can be used to quantify changes in mechanical properties before and after cleaning [28,31]. Dynamic mechanical analysis (DMA) provides additional insights into viscoelastic properties, including storage modulus (elastic response) and loss modulus (viscous response), which are sensitive indicators of polymer chain degradation and fiber embrittlement [48]. Zero-span tensile testing, which measures fiber strength independent of fiber-fiber bonding by reducing the clamping distance to nearly zero, is particularly useful for assessing the intrinsic strength of cellulose fibers after cleaning treatments [51].

For metal artifacts, physical evaluation of cleaning outcomes includes measurements of corrosion recurrence rate (e.g., area percentage of new corrosion products over time, measured via digital image analysis), surface roughness before and after cleaning, and mass loss (mg/cm^2^) due to corrosion or cleaning. Long-term monitoring of corrosion recurrence is particularly important, as incomplete removal of chlorides or other corrosive agents can lead to post-cleaning degradation within months or years. Accelerated aging tests (e.g., exposure to controlled humidity at 80–90% RH for 4–8 weeks) can predict long-term stability, with lower post-cleaning corrosion rates indicating more durable cleaning outcomes [18,20].

Furthermore, physical cleaning methods such as ultrasonic cleaning and micro–nano-bubble techniques can remove contaminants while preserving delicate substrates, offering critical guidance for method selection [40,44]. The mechanical effects of cleaning can also be evaluated using atomic force microscopy (AFM) to measure surface stiffness and adhesion forces at the nanoscale, providing complementary data to macroscopic mechanical tests [28].

### 4.2. Chemical Analyses (Quantifiable and Semi-Quantitative)

Chemical evaluation detects residual contaminants and changes in the material composition of the substrate. Quantifiable chemical indicators include QCM-D mass removal rates (μg/cm^2^), leaching concentrations (mg/L), and degradation product yields (%). Semi-quantitative methods such as FTIR peak intensity ratios and NMR signal integration provide comparative data without absolute calibration.

Common analytical tools include Fourier-transform infrared spectroscopy (FTIR), nuclear magnetic resonance (NMR), mass spectrometry, and elemental analysis [14]. These techniques can reveal whether cleaning agents or nanofluids penetrate the substrate and whether organic or inorganic components have been leached or degraded [20,37].

FTIR spectroscopy is widely used to identify functional groups and monitor chemical changes in heritage materials. Attenuated total reflection (ATR)-FTIR enables surface-specific analysis without sample preparation, making it ideal for assessing the removal of organic contaminants and detecting residual cleaning agents. For quantitative or semi-quantitative analysis, FTIR peak intensity ratios (e.g., contaminant peak / reference peak) can track removal progress, while spectral subtraction methods quantify residual cleaning agents by comparing treated and untreated reference spectra. Micro-FTIR mapping can generate chemical images showing the spatial distribution of contaminants and cleaning agents across the artifact surface [20].

Pyrolysis-gas chromatography/mass spectrometry (Py-GC/MS) provides detailed molecular information about complex organic mixtures, including natural resins, waxes, oils, and synthetic polymers. Quantitative Py-GC/MS can determine the absolute concentration of specific compounds (mg/g artifact) using internal standards, enabling precise measurement of contaminant removal efficiency. This technique is particularly valuable for evaluating the removal of aged coatings and adhesives, as it can identify degradation products and assess the selectivity of cleaning treatments [20].

Quartz Crystal Microbalance with Dissipation (QCM-D) is a fully quantitative method that allows real-time monitoring of polymer coating mass changes and removal kinetics during nanofluid cleaning, enabling dynamic and quantitative assessment [49]. QCM-D provides simultaneous measurements of mass uptake/release (frequency shift, Δf, in Hz, convertible to ng/cm^2^) and viscoelastic properties (dissipation shift, ΔD), offering insights into the swelling, softening, and detachment mechanisms of polymer coatings during cleaning [35]. The removal rate (ng/cm^2^/s) can be calculated from the slope of the frequency change over time, providing a direct kinetic parameter for comparing cleaning efficacy across different formulations.

### 4.3. Optical Characterization (Quantitative and Semi-Quantitative)

Optical methods, including colorimetric analysis, microscopy, and laser scanning, provide direct visual feedback on cleaning efficacy and surface quality [11,52].

**Figure 6:**
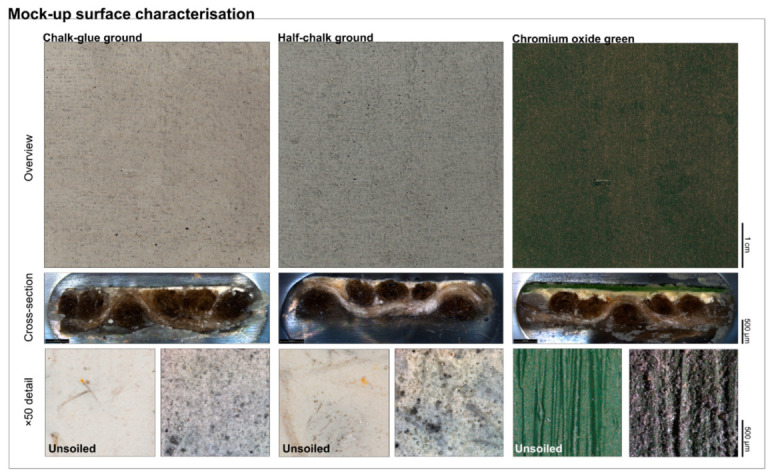
Overview (top row), cross-sections (central row), and magnified detail (bottom row) of the soiled mock-up surfaces (5 cm × 5 cm) cleaned during this study (unsoiled mock-ups are shown for comparison purposes, indicating the degree of soiling and differences in surface topography). The three mock-up classes included a chalk-glue ground, a half-chalk ground, and a chromium oxide green oil paint in linseed oil applied onto a half-chalk ground [51].

Colorimetric analysis (ΔE) is a fully quantitative method that quantifies the changes in surface color before and after cleaning by assessing contaminant removal and pigment preservation [51]. An example of a colorimetric evaluation applied to mock-up cleaning experiments is shown in Figure 6.

Color differences (ΔE) are calculated using the Euclidean distance formula according to the CIE 1976 L*a*b* colour space. According to CIE Publication No. 116, ΔE values below approximately 1.0 are considered imperceptible to the average human observer, while values above 3.0 indicate clearly perceptible colour differences [53].

Multispectral imaging (MSI) and hyperspectral imaging (HSI) extend colorimetric analysis by capturing reflectance spectra across multiple wavelength bands (e.g., ultraviolet, visible, and near-infrared). These techniques provide semi-quantitative data, as reflectance values at specific wavelengths can be compared before and after cleaning (e.g., % reflectance increase at 550 nm), enabling mapping of contaminant distribution and assessment of cleaning uniformity [54].

Microscopy methods (SEM, confocal laser microscopy, optical microscopy) provide valuable visual information but are primarily qualitative or semi-quantitative. SEM can measure surface feature dimensions (e.g., particle size, crack width in micrometers) and enable quantitative element analysis via EDX (wt% or at%). However, overall cleaning assessment remains observer-dependent unless systematic scoring criteria are defined. Therefore, these methods are most appropriately classified as semi-quantitative, best used in combination with fully quantitative techniques [50].

Confocal laser scanning microscopy (CLSM) provides three-dimensional surface profiling with sub-micrometer resolution, enabling quantitative assessment of surface roughness (Ra, Rq, and Rmax in μm), porosity (%), and cleaning-induced topographical changes [50].

### 4.4. Multidimensional Integrated Evaluation Framework

By integrating physical, chemical, and optical indicators, a multidimensional quantitative evaluation framework can be constructed, enabling quantitative and comparative analysis of cleaning outcomes and inter-method performance differences within a unified evaluation structure [11,49].

A key extension of this framework is the inclusion of long-term stability indicators, which assess post-treatment durability:

- Contaminant recurrence rate (stone): area percentage of re-soiling over time;
- Mechanical property retention rate (paper/textile): strength retention after aging;
- Corrosion recurrence rate (metals): new corrosion area under humid aging;
- Microbial recolonization rate: ATP bioluminescence (RLU).

These indicators extend evaluation beyond immediate cleaning performance to long-term conservation stability.

Multivariate statistical tools such as PCA and PLSR integrate multi-source data for pattern recognition and correlation analysis.

### 4.5. Sustainability Metrics and Environmental Impact Assessment

Under the green cleaning paradigm, evaluation frameworks should also account for environmental and operator safety metrics, including cleaning agent consumption, solvent volatilization, and waste disposal safety [48,55].

For nanomaterials and nanofluids, leaching behavior should be quantified using immersion tests, with release levels expressed in μg/cm^2^ or mg/L. Ecotoxicity evaluation using model organisms (e.g., Daphnia magna and Vibrio fischeri) is required to assess environmental safety.

Life Cycle Assessment (LCA) provides a full-system evaluation from production to disposal. Studies show that nanofluid-based cleaning can reduce solvent consumption by up to 90% compared to conventional methods [55].

Green chemistry metrics such as the E-factor and atom economy are also used to evaluate sustainability efficiency.

Post-treatment monitoring is a critical component of sustainable evaluation. Delayed degradation phenomena—such as salt recrystallization, enzyme reactivation, microbial recolonization, and corrosion recurrence—require follow-up assessments at 1, 6, and 12 months.

Importantly, this monitoring strategy is defined strictly as an evaluation and documentation procedure, not a decision-making or optimization tool.

### 4.6. Limitations of Current Evaluation Systems

Despite progress, current evaluation systems still face several limitations:

- Lack of standardized thresholds across studies;
- Poor inter-study comparability of quantitative indicators;
- Insufficient long-term monitoring datasets;
- Over-reliance on short-term surface appearance assessment.

These limitations highlight the need for unified evaluation standards and long-term data-driven conservation frameworks.

## 5. Conclusions

This review summarizes recent advances in the cleaning of cultural heritage artifacts, encompassing surface materials and deterioration mechanisms, traditional and advanced cleaning methods, multidimensional quantitative evaluation, and sustainable strategies. Different materials exhibit significant variability in contaminant adsorption and sensitivity to cleaning, highlighting the necessity for material-specific cleaning approaches. Modern techniques have evolved from single physical or chemical methods to integrated, multidimensional, selective, and controllable strategies, including nanofluids, microemulsion gels, functional hydrogels, enzyme-assisted cleaning, and laser-based methods. These approaches enhance cleaning efficiency and selectivity while minimizing damage to the substrate and incorporating environmentally friendly practices.

Quantitative evaluation systems integrating optical, colorimetric, mechanical, chemical, and biological indicators provide a scientific basis for assessing both cleaning effectiveness and substrate safety. A critical insight emerging from current research is that immediate cleaning effectiveness does not guarantee long-term stability. Therefore, the assessment of post-treatment durability—including contaminant recurrence rate, material property retention rate, corrosion recurrence, and microbial recolonization—should be an integral component of any cleaning evaluation framework. Sustainable strategies further ensure minimal risk to both operators and the environment.

Key principles emerging from current research include selective cleaning, multidimensional evaluation, green and sustainable methods, integration of multiple techniques, and—crucially—the emphasis on long-term stability as a core objective of cleaning interventions.

Future studies will benefit from incorporating a broader range of recent literature in heritage science, conservation chemistry, and materials science. Recent publications in heritage science, conservation chemistry, and materials science provide a rich body of evidence supporting the trends and recommendations outlined above [29,55].

Collectively, these advancements point toward selective, quantifiable, controllable, and durable cleaning strategies for cultural heritage artifacts.

## Figures and Tables

**Table 1:** Overview of surface cleaning methods for cultural heritage artifacts.

Cleaning Type	Typical Methods	Applicable Artifacts	Mechanism of Action	Advantages	Limitations	Key References
Chemical Cleaning	Solvents, microemulsions, nanofluids	Oil, resin, or polymer-based contaminants	Dissolution, emulsification, interface energy reduction	Efficient, selective	Potential substrate infiltration; requires parameter optimization	[5,13,34,35,36]
Physical Cleaning	Laser cleaning, ultrasonic cleaning, micro-nano bubbles	Painted, paper, metal, fiber-based artifacts	Photothermal effect, cavitation, shock waves	Non-contact, high selectivity, green	Sensitive to energy density and wavelength; precise control needed	[18,37,38,39,40]
Enzyme/Microbial	Enzyme gels, microbial cleaning	Protein, cellulose, lipid contaminants	Enzymatic hydrolysis of pollutants	Eco-friendly, gentle, selective	Slow action; suitable for localized or combined use	[21,30,41,42,43,44]

## Data Availability

No new data were created or analyzed in this study.
